# Catalogue of epidermal genes: Genes expressed in the epidermis during larval molt of the silkworm *Bombyx mori*

**DOI:** 10.1186/1471-2164-9-396

**Published:** 2008-08-22

**Authors:** Shun Okamoto, Ryo Futahashi, Tetsuya Kojima, Kazuei Mita, Haruhiko Fujiwara

**Affiliations:** 1 Department of Integrated Biosciences, Graduate School of Frontier Sciences, The University of Tokyo, Kashiwa, Chiba 277-8562, Japan; 2 Division of Insect Sciences, National Institute of Agrobiological Sciences, Tsukuba, Ibaraki 305-8643, Japan

## Abstract

**Background:**

The insect cuticle is composed of various proteins and formed during the molt under hormonal regulation, although its precise composition and formation mechanism are largely unknown. The exhaustive catalogue of genes expressed in epidermis at the molt constitutes a massive amount of information from which to draw a complete picture of the molt and cuticle formation in insects. Therefore, we have catalogued a library of full-length cDNAs (designated epM) from epidermal cells during the last larval molt of *Bombyx mori*.

**Results:**

Of the 10,368 sequences in the library, we isolated 6,653 usable expressed sequence tags (ESTs), which were categorized into 1,451 nonredundant gene clusters. Seventy-one clusters were considered to be isoforms or premature forms of other clusters. Therefore, we have identified 1,380 putative genes. Of the 6,653 expressed sequences, 48% were derived from 92 cuticular protein genes (RR-1, 24; RR-2, 17; glycine-rich, 29; other classes, 22). A comparison of epM with another epidermal EST data set, epV3 (feeding stage: fifth instar, day 3), showed marked differences in cuticular protein gene. Various types of cuticular proteins are expressed in epM but virtually only RR-1 proteins were expressed in epV3. Cuticular protein genes expressed specifically in epidermis, with several types of expression patterns during the molt, suggest different types of responses to the ecdysteroid pulse. Compared with other *Bombyx *EST libraries, 13 genes were preferentially included in epM data set. We isolated 290 genes for proteins other than cuticular proteins, whose amino acid sequences retain putative signal peptides, suggesting that they play some role in cuticle formation or in other molting events. Several gene groups were also included in this data set: hormone metabolism, P450, modifier of cuticular protein structure, small-ligand-binding protein, transcription factor, and pigmentation genes.

**Conclusion:**

We have identified 1,380 genes in epM data set and 13 preferentially expressed genes in epidermis at the molt. The comparison of the epM and other EST libraries clarified the totally different gene expression patterns in epidermis between the molting and feeding stages and many novel tissue- and stage-specifically expressed epidermal genes. These data should further our understanding of cuticle formation and the insect molt.

## Background

Insect cuticles are formed during molting under hormonal regulation and are composed of complex and composite materials, made mainly of chitinous filaments embedded in proteinaceous layers (see [1] for review). The cuticle acts as both skin and exoskeleton, and has diversified in its mechanical properties for optimal biological functions in various insects. The differences in the mechanical properties of the exoskeleton are probably dependent on the respective features of various cuticular proteins and chitin itself, on the precise combination of different cuticular proteins, and on their secondary stabilization, called "sclerotization". However, the number of types of cuticular proteins included in the cuticle has not yet been resolved, nor how their coordinated expression in epidermal cells and their excretion into the cuticle are precisely controlled during molting. These issues are very important for any understanding of the mechanisms underlying the formation of the highly ordered and layered structure of the cuticle.

The amino acid sequences of cuticular proteins have been reported for a wide variety of insects, by directly sequencing the purified cuticular proteins or by their deduction from the corresponding cDNA sequences [1-3]. However, most excreted cuticle components are cross-linked, making them unextractable [2]. Therefore, we infer that many other cuticular proteins are yet to be identified. Previously reported information on cuticular protein sequences has revealed several conserved motifs, such as the R&R consensus [1], Tweedle [4], and a 44-amino-acid motif [5]. The R&R consensus sequence is the most prevalent motif, and was first reported by Rebers and Riddiford [6]. An extended version of this consensus sequence was subsequently described and is generally referred to as the R&R consensus, which is known to bind chitin [1,7,8]. Three distinct forms of this consensus are recognized: RR-1, RR-2, and RR-3 [1,9]. RR-1 is characteristic of proteins in soft and flexible cuticles, and RR-2 proteins are associated with stiff and hard cuticles in general, although this classification is tentative [1,10]. Many other cuticular proteins lacking these motifs have structures containing other repeated structures, such as GGX or AAP(A/V) [1,11,12].

The comprehensive identification of cuticular proteins with an R&R motif has been attempted in *Drosophila melanogaster *[13], *Apis mellifera *[14], and *Anopheles gambiae *[15], based on their genome sequences. Recently, we identified 220 putative cuticular protein genes (RR-1 56, RR-2 89, RR-3 3, Tweedle 4, CPF 1, CPFL 4, glycine-rich 29, and 34 other genes) in the silkworm *Bombyx mori *[12]. A key question is whether each cuticular protein gene is expressed in a tissue- or stage-specific manner. Togawa et al. [16] investigated the developmental expression patterns of all *Anopheles *cuticular protein genes with R&R consensus motifs, using real-time quantitative reverse transcription-polymerase chain reaction (qRT-PCR). They found that all the genes are expressed and can be grouped into 21 clusters with different developmental expression profiles, although their tissue specificities were not examined.

*Bombyx *has an advantage in the study of tissue specificity and the expression profiles of each tissue because its tissues are relatively large and it is easy to construct a tissue-specific cDNA library. More than 40 expressed sequence tag (EST) databases for various tissues at the different developmental stages of *Bombyx *are available [17-19]. Exhaustive sequencing of the full-length cDNAs expressed in epidermal cells is an effective method of gaining an overview of cuticular proteins, because it avoids directly sequencing the barely extractable cuticular proteins. Therefore, we constructed a library of full-length cDNAs, using a G-capping method [20], from mRNAs expressed in the larval epidermal cells of the silkworm, *B. mori*, during the fourth larval molt, when the subsequent cuticle of the fifth instar is formed.

We sequenced over 10,000 clones randomly selected from the library described above, and isolated 6,653 ESTs belonging to 1,451 nonredundant gene clusters. Seventy-one clusters were considered to be isoforms or premature forms of other clusters. Therefore, we identified 1,380 putative genes. About half of these ESTs encoded cuticular proteins, representing 92 genes. In addition to cuticular proteins, we also identified 290 genes encoding the amino acid sequences of their putative signal peptides, suggesting that they play some role in cuticle formation or other molting events. Here, we list those cuticular protein genes, secreted protein genes, and other epidermal protein genes, including those encoding transcription factors and many enzymes. These data should be useful in understanding cuticle formation and the insect molt.

## Results & Discussion

### Construction and characterization of the epM EST data set

**Figure 1:**
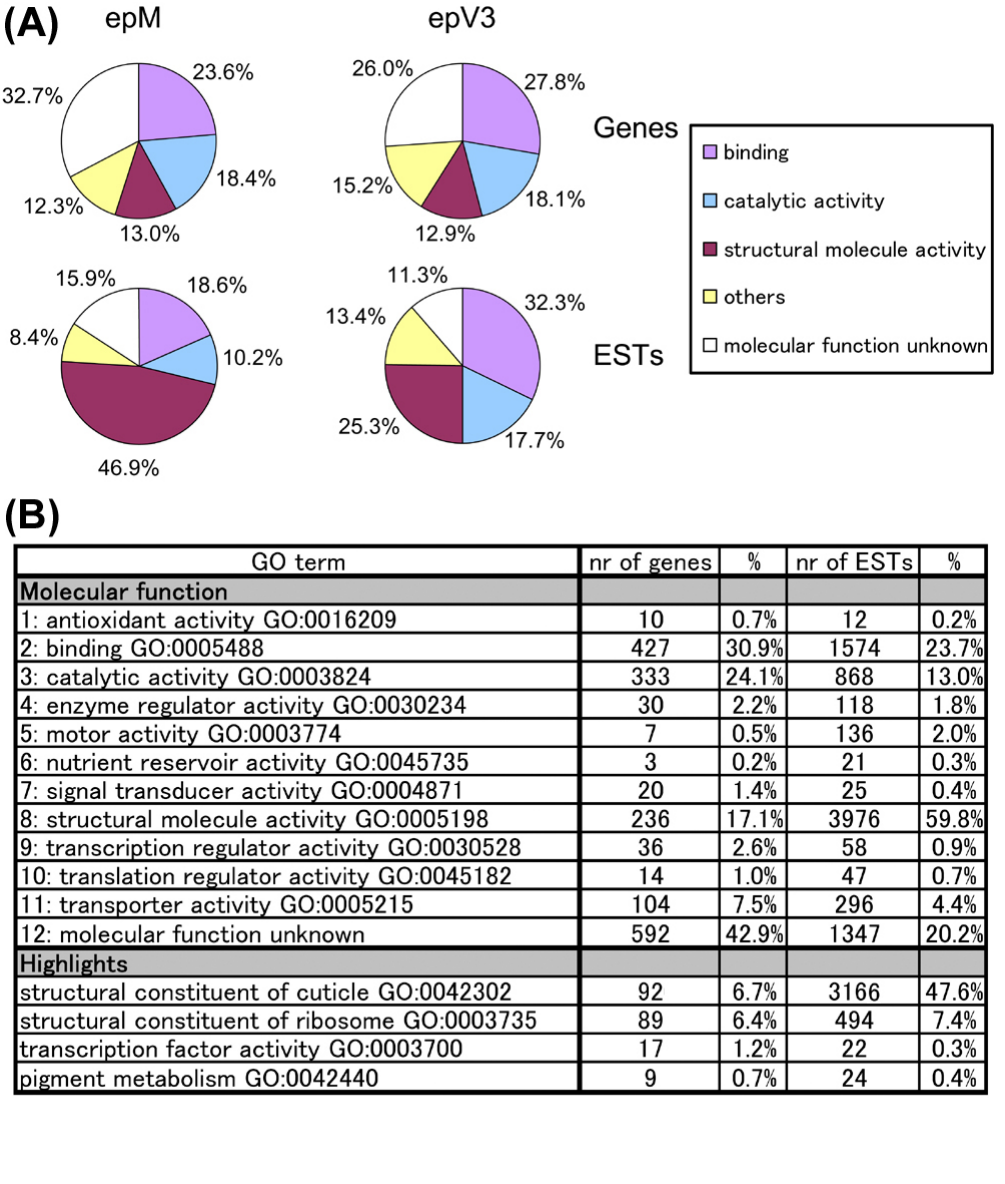
**Assignment of Gene Ontology (GO) molecular function terms to the two epidermal EST data sets of *Bombyx mori***. (A) The ratio of gene numbers (upper) and total EST clones (lower) of the representative 4 GO terms are shown. (B) The ratio of genes classified into each GO term. The percentage of gene numbers (left) and total EST clones (right) were shown. Because one cluster can be associated with more than one GO term and total percentages summing all categories are shown as 100 in (A), percentage in (B) is different from that in (A). Highlights show the characteristic gene families such as cuticular proteins and transcription factors.

To exhaustively identify the cuticular protein components and epidermal genes expressed during the larval molting stages of the silkworm, we constructed a library of full-length cDNAs by mixing the RNAs of eight consecutive stages (C1, C2, D1, D2, D3, E1, E2, and F, according to [20]). We designated this cDNA library epM (fourth instar **ep**idermis in **M**olting). We randomly sequenced 10,368 clones, excluded any short insert sequences, and isolated 6,653 ESTs (GenBank accession numbers DC432783–DC439435; Table 1, Additional File 1). These ESTs were classified into 1,451 nonredundant EST clusters/singletons. Seventy-one clusters were considered to be isoforms or premature forms of other clusters (Additional File 2). Excluding these 71 clusters, we identified 1,380 putative gene clusters, 885 of which were singletons (only one clone included in the EST data set). Among the 1,380 gene clusters, 1,025 clusters had sequence similarities to *Drosophila *genes (*P *< 1e-05). We categorized the gene clusters of epM using the criteria for the gene ontology (GO) terms used for *Drosophila *[21] (Figure 1). Figure 1B shows the numbers of gene cluster types (number of genes; left column), some of which are categorized by multiple criteria, and the numbers of total EST clones included under those criteria, which represent their levels of expression (number of ESTs; right column). Figure 1A shows several major categories in the same ratios as shown in Figure 1B, but the total percentages, summing all categories, are shown as 100 in Figure 1A (total epM genes 131%, total epM ESTs 127%, in Figure 1B). It is remarkable that structural protein genes are expressed so abundantly (60% of the total ESTs) (Figure 1A, 1B, category 8), many of which encode cuticular proteins (Figure 1B, highlights; 48% of total ESTs), which suggests the active translation of cuticle components in the epidermal cells during the molt (see below).

**Table 1:** Summary of epM EST data set

Number of sequenced bacterial clones	10,368
Number of ESTs	6,653
Number of putative genes (nonredundant clusters)*1	1,380 (1,451)
Number of genes with similarity to *Drosophila *genes *2	1,025

*1: 71 clusters were considered to be isoforms/premature forms of other clusters.

*2: The threshold maximum E-value was set to 1e-05 by Blastx search.

Following the structural protein genes, the genes in the categories "binding activity" (Figure 1B, category 2) and "catalytic activity" (Figure 1B, category 3) were the most abundant, in both the number of species (31% and 24% genes, respectively) and the level of expression (24% and 13%, respectively) (Figure 1B). Considerable numbers of molecules in these categories are thought to be involved in the formation of the cuticle structure during the molt. In the molting stages, new cuticle is synthesized and constructed to replace the old cuticle during its rapid apolysis. More than 30 species of proteolytic molecules (see below), ubiquitin cascades, and various degradation pathways are involved in the apolysis of the old cuticle structure. In contrast, many enzymes involved in glycolysis, ATP synthesis, and electron transport produce the energy for the amino acid metabolism and protein synthesis required for new cuticle formation. Chitin-binding proteins, phenoloxidase-activating enzymes, and laccase are also involved in the modification of cuticular proteins (sclerotization), and signal peptidase and several protein transporters are involved in the transport of cuticular components (Additional File 3). We also found putative genes related to insect hormone metabolism, small-ligand binding, and transcription (Additional File 3), which are summarized below.

**Figure 2:**
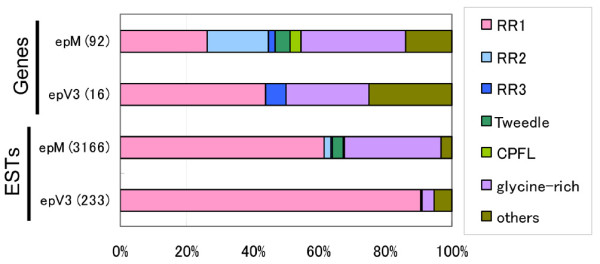
**Composition of cuticular protein genes in two epidermal EST libraries (epM, epV3)**. The percentages of gene numbers (upper) and total clone numbers (lower) of each motif are shown. The numbers of gene numbers and total ESTs are shown in parentheses.

A comparison of the categorized gene clusters of epM and epV3 shows that the percentage of structural protein genes expressed in epM is much larger than that in epV3 (Figure 1A), and that more different types of cuticular protein genes are represented in epM than in epV3 (Figure 2). This observation suggests that many types of cuticular proteins are involved in cuticle formation in the molting stages, whereas the intermolt cuticle is built of only a few kinds of cuticular proteins (most of them are class RR-1 proteins; Figure 2), which may contribute to the thickening of the endocuticle layer.

### Species, structure, and expression of cuticular proteins encoded by genes in epM

**Figure 3:**
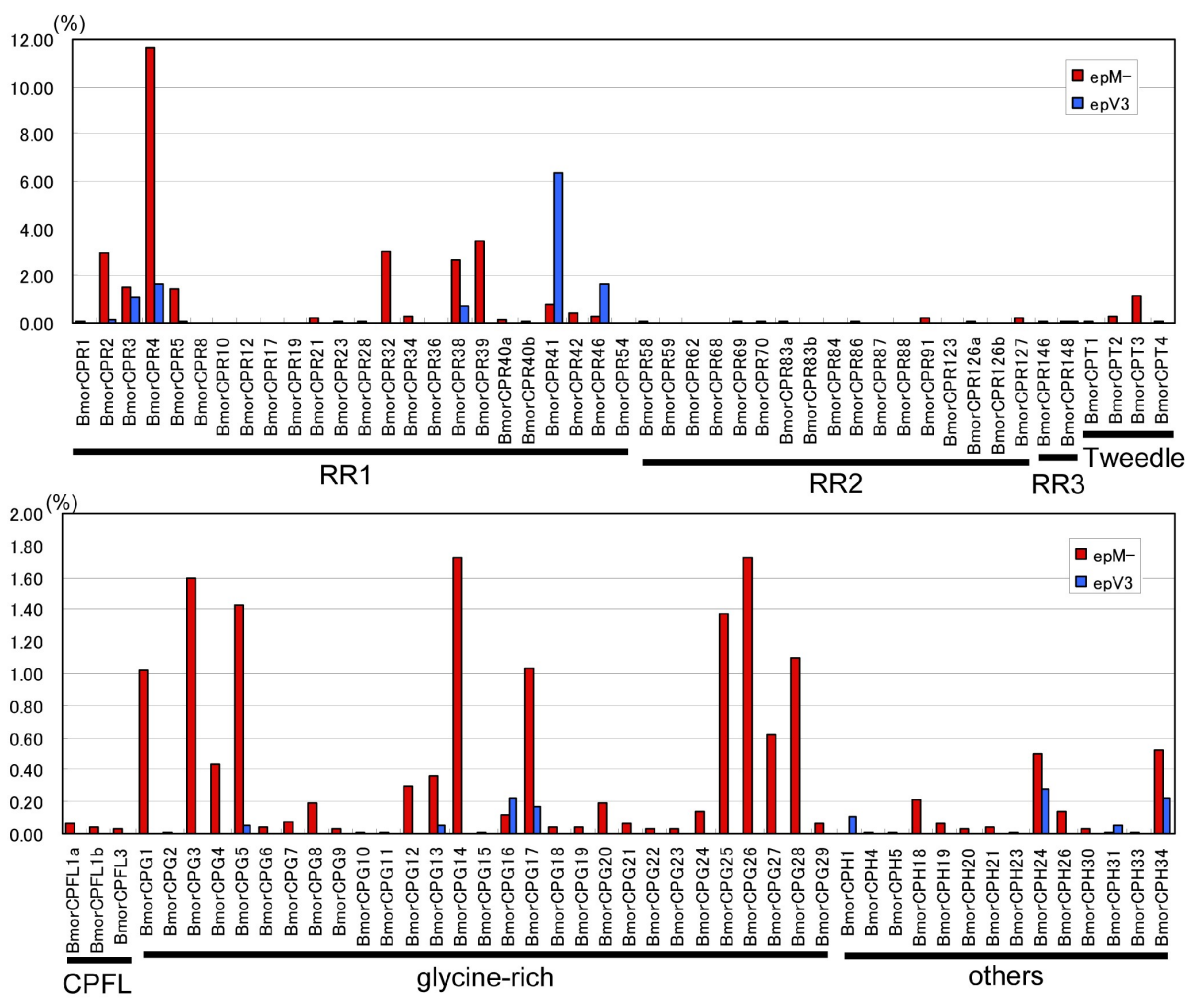
**The ratio (ESTs of each gene/total ESTs) of each cuticular protein gene in two *Bombyx *epidermal ESTs**. Red columns indicate the ratio of epM library and blue columns indicate that of epV3 library. Cuticular motif of each gene was also shown below the lines.

We identified 92 cuticular protein genes in the epM library (Figures 2 and 3, Additional File 4). These genes correspond to 88 cuticular protein genes identified in the *Bombyx *p50 strain [12]. In four cases (*BmorCPFL1*, *BmorCPR40*, *BmorCPR83*, and *BmorCPR126*), two different genes in the epM data set corresponded to the same gene of the p50 strain. Because we have repeatedly mated the +^p ^strain with its p^S ^sibling (sibmating), the two strains have similar genetic backgrounds. Therefore, the sequence variations identified in this study may result from the differences between +^p ^(or p^S^) and p50. It has been reported that the copy numbers of cuticular protein genes vary even among strains of *D. melanogaster *[22] and *A. gambiae *[15], suggesting that the copy numbers of cuticular protein genes also vary among *Bombyx *strains in the four cases cited above, although we cannot exclude the possibility that sequence differences are the result of strain differences between the +^p ^and p^S ^strains.

Transcripts corresponding to 43 cuticular proteins in the epM cDNA library are predicted to have the R&R consensus motif in their amino acid sequences (Figure 3, Additional File 4). Twenty-four were RR-1, 17 were RR-2, and two were RR-3 proteins. A comparison of the R&R protein transcripts in epM and epV3 demonstrated totally different expression patterns. In the molting stages (epM), transcripts for the 24 RR-1 protein genes comprised 61% of the total ESTs for cuticular protein genes. However, in the intermolt of the fifth instar stages (epV3), only seven RR-1 protein transcripts comprised 91% of the total ESTs of the cuticular protein genes (Figure 2). In contrast, 17 RR-2 protein transcripts were found in epM (2.0% of the total cuticular protein ESTs), whereas no RR-2 protein transcripts were found in epV3 (Figure 2, Additional File 4). Cox and Willis [23] reported that the protein composition of the cuticle correlates with the flexibility of the mature cuticle. We found that RR-1 protein genes were abundantly expressed in epM compared with their expression in other EST libraries [12], which is consistent with the general claim that RR-1 proteins are involved in the flexible cuticle. The dominant expression in epV3 of RR-1 proteins, which affect the thickness of the endocuticle region during the intermolt, may contribute to the flexible cuticle structure in the feeding stages. The lack of RR-2 protein genes in epV3 suggests that these proteins are mainly involved in the formation of the outer cuticle region, the exocuticle or epicuticle layers, during the molting stages. It is noteworthy that transcripts corresponding not only to R&R proteins but also to other types of cuticular proteins (see below) are abundant in the epM library (Figure 2), and may therefore be essential for the formation of new cuticle.

### Glycine-rich cuticular protein genes

As well as those with R&R consensus motifs, cuticular proteins with the glycine-rich motif (CPG) are often observed in *Bombyx *[12,24,25]. We found 29 types of CPG transcripts in the epM library (29% of the total cuticular protein genes; Figure 2). In the epV3 library, only four CPG protein transcripts were found, and at lower levels (3.9% in total; Figure 2). Andersen et al. [2] predicted that Gly-Gly (GG) repeats would form turn structures in proteins, and some proteins contain glycine-rich regions that include GG repeats [24-27]. We previously reported that the expression of the cuticular protein BMCPG1 (BmorCPG1 in this study), which has many Gly-Gly-Tyr (GGY) repeats and sequence similarity to *Drosophila *EDG91, is dependent on the ecdysteroid pulse during the fourth molt [26]. We also found that the tyrosine residues of GGY repeats were cross-linked to di-DOPA by tyrosinase treatment [28] (Fujiwara et al., in preparation), suggesting that the GGY motif is involved in protein cross-linking during sclerotization [29].

A comparison with other EST libraries showed that 12 CPG genes (*BmorCPG2*, *BmorCPG3*, *BmorCPG7*, *BmorCPG8*, *BmorCPG15*, *BmorCPG19*, *BmorCPG21*, *BmorCPG23*, *BmorCPG25*, *BmorCPG26*, *BmorCPG27*, and *BmorCPG28*) are specifically expressed in the epM library (Additional File 1). Transcripts of the CPG proteins in other libraries were found mainly in epithelial cells, such as the antenna, compound eye, and wing disc, suggesting that CPG proteins are components of body surfaces in general [12]. It is interesting that CPG proteins are usually positively charged, with a high isoelectric pH (> 8.0), and that these features are not observed in other cuticular proteins. This property of CPG proteins may contribute to their physical interaction with other types of cuticular proteins and between CPGs in the cuticle layer.

### Other cuticular protein genes

Recently, other types of cuticular protein genes have been reported. Tweedle proteins, which are suggested to interact directly with chitin, are observed in the epidermis, tracheal tree, foregut, and wing discs in *D. melanogaster *[4]. There are four Tweedle protein genes in *Bombyx *(*BmorCPT1*-*BmorCPT4*) [12], and transcripts of all four Tweedle genes are found in the epM library (3.3% of the total cuticular protein genes), but are not present in the epV3 library (Figure 2). Togawa et al. [5] reported a cuticular protein with a 44-amino-acid motif (CPF) and CPF-like proteins (CPFL). There are one CPF and four CPFL genes in the *Bombyx *p50 strain [12], and we found three CPFL protein transcripts in the epM library (two of them are very similar and correspond to the gene *BmorCPFL1 *in the p50 strain). These CPFL protein transcripts occur in small amounts in epM (0.3%), whereas they are not present at all in the epV3 EST data set (Figures 2 and 3).

In this study, we also found 13 hypothetical cuticular proteins (CPH, Additional File 4) [12]. Some CPHs have the conserved motif. BmorCPH30 and BmorCPH31 have an 18-residue motif (PV)xDTPEVAAA(KR)AA(HF)xAA(HY), and seven CPH proteins (BmorCPH18, BmorCPH19, BmorCPH21, BmorCPH23, BmorCPH24, BmorCPH26, and BmorCPH34) have the AAP(A/V) motif, suggesting common roles in cuticle formation. However, CPH proteins have various structural features, amino acid compositions, repeated structures, and isoelectric points, so we cannot summarize their functional roles at present.

### Highly expressed cuticular protein genes in the epM data set

Table 2 shows the top 50 genes most abundantly expressed in the epM library. Of these 50 genes, 29 are cuticular protein genes. *BmorCPR4 *(formally known as *LCP18*) is the most abundantly expressed (776 clones corresponding to 11.7% of the total epM ESTs and 24.5% of the epM cuticular protein transcripts), followed by *BmorCPR39*, *BmorCPR32*, *BmorCPR2 *(formally known as *LCP17*), and *BmorCPR38 *(formally known as *LCP22*). *BmorCPR4 *is also expressed abundantly in epV3 (30 clones; 12.9% of the epV3 cuticular protein transcripts), which is consistent with the former observation that this gene is also expressed in the intermolt stage [30]. BmorCPR4, the main component of the larval cuticle, is suggested to be orthologous to *Hyalophora cecropia *HCCP12 and *Manduca sexta *MSCP14.6 [30]. Similarly, BmorCPR38 and BmorCPR3 (5.7% and 3.1% of the epM cuticular protein transcripts, respectively) are abundantly expressed not only in epM but also in epV3 (5.6% and 8.6% of the epV3 cuticular protein transcripts, respectively; Figure 3). These cuticular proteins are constitutively expressed during both the molt and intermolt stages. In contrast, BmorCPR41 (formally known as LCP30) and BmorCPR46 (formally known as Bmwcp11) are more abundantly expressed during the intermolt stages (49.4% and 12.9% of the epV3 cuticular protein transcripts, respectively) than during the molt (1.7% and 0.6% of the epM cuticular protein transcripts, respectively). *BmorCPR41 *and *BmorCPR46 *comprise more than 62% of the cuticular protein transcripts in epV3, and these are therefore intermolt-specific genes. This observation is consistent with the fact that *BmorCPR46 *is mainly expressed at the intermolt stage in the wing disc [12].

**Table 2:** Top 50 EST genes

Cluster No.	Total ESTs	Gene Name	*Drosophila *CG Number	e-value*	score*
1	776	BmorCPR4	CG32405	2E-24	108
2	230	BmorCPR39	CG30045	6E-25	111
3	202	BmorCPR32	CG30045	1E-39	159
4	195	BmorCPR2	CG30045	2E-26	115
5	180	BmorCPR38	CG30045	9E-25	110
6	116	-	-	-	-
7	115	BmorCPG26	CG14191	6E-22	100
8	115	BmorCPG14	CG2150	1E-21	100
9	106	BmorCPG3	CG15597	2E-22	102
10	100	Actin 57B	CG10067	0	756
11	99	BmorCPR3	CG30045	2E-25	112
12	97	BmorCPR5	CG32405	3E-20	94.7
13	95	BmorCPG5	CG14191	7E-18	87.4
14	91	BmorCPG25	CG13050	6E-13	70.9
15	87	Myosin light chain 2	CG2184	7E-69	257
16	76	BmorCPT3	CG5812	5E-83	305
17	73	BmorCPG28	CG15597	9E-20	93.2
18	69	BmorCPG17	CG5225	7E-08	53.5
19	68	BmorCPG1	CG14191	4E-24	107
20	63	Tropomyosin 1	CG4898	9E-140	493
21	60	CG32774	CG32774	1E-06	50.8
22	53	BmorCPR41	CG30042	1E-30	130
23	47	CG13731	CG13731	2E-19	94.4
24	41	BmorCPG27	CG14191	5E-21	97.4
25	35	Troponin C at 73F	CG7930	3E-65	244
26	35	BmorCPH34	CG34333	7E-33	137
27	35	wings up A	CG7178	2E-98	355
28	34	Myosin alkali light chain 1	CG5596	2E-50	196
29	33	BmorCPH24	CG32564	4E-10	61.2
30	31	-	-	-	-
31	29	lethal (2) essential for life	CG4533	3E-55	211
32	29	BmorCPG4	CG32564	1E-37	154
33	28	BmorCPR42	CG7658	2E-21	99
34	24	BmorCPG13	CG30101	2E-91	333
35	24	mitochondrial ATPase subunit 6	CG34073	4E-51	198
36	22	Muscle LIM protein at 60A	CG33149	8E-41	163
37	22	upheld	CG7107	3E-171	598
38	22	-	-	-	-
39	21	BmorCPT2	CG5812	8E-70	261
40	20	Ejaculatory bulb protein III	CG11390	2E-24	109
41	20	BmorCPR46	CG2555	3E-16	81.6
42	20	BmorCPG12	CG16886	2E-66	250
43	19	BmorCPR34	CG8515	7E-42	168
44	18	Kaz1-ORFB	CG1220	4E-12	67.8
45	18	Gasp	CG10287	6E-137	484
46	18	CG31997	CG31997	2E-48	189
47	17	Tropomyosin 2	CG4843	2E-131	465
48	17	Translationally controlled tumor protein	CG4800	3E-78	288
49	17	Ribosomal protein S20	CG15693	1E-51	198
50	15	BmorCPR21	CG12330	2E-23	106
51	15	CG17127	CG17127	4E-31	131
52	15	Ribosomal protein LP2	CG4918	4E-39	157
53	15	BmorCPR127	CG13935	4E-36	148
54	15	Ribosomal protein L5	CG17489	2E-137	485
55	15	LDLa domain containing chitin binding protein 1	CG8756	1E-112	404

* E-value and score are calculated in *Drosophila *homologs.

As shown in Figure 3, most cuticular protein genes are molt specific. The second most abundantly expressed, *BmorCPR39 *(7.3% of the epM cuticular protein transcripts), and the third most abundantly expressed, *BmorCPR32 *(6.4% of the epM cuticular protein transcripts), of the epM ESTs were not found in epV3 (Additional File 4). Of the 29 cuticular protein genes in the list of the 50 most abundantly expressed genes (Table 2), 17 are not found in epV3 (Figure 3, Additional File 4). The fact that many molt-specific cuticular protein genes are identified here supports the effectiveness of the cDNA-based screening of cuticle components.

### Temporal and tissue-specific expression patterns of major cuticular protein genes during the molt

**Figure 4:**
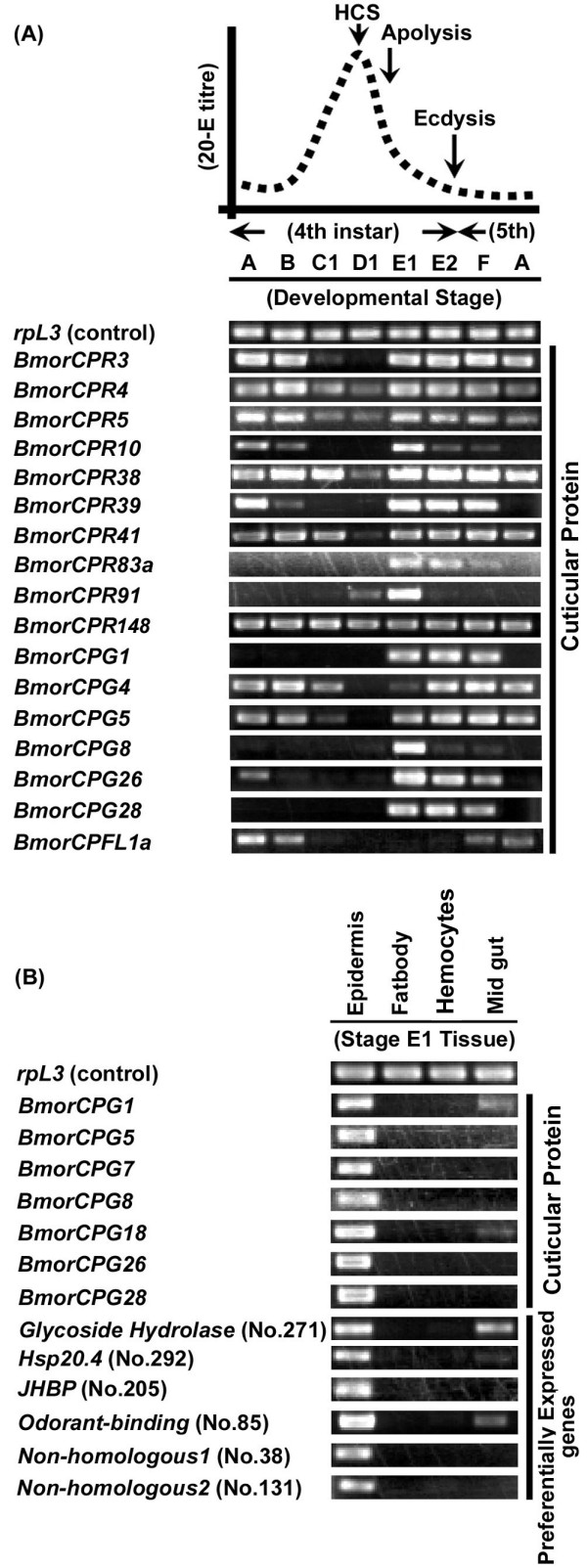
**Stage and Tissue specific expression of epidermal genes isolated in epM data set by RT-PCR**. (A) Expression profile of several cuticular protein genes by RT-PCR analysis. Relative hemolymph ecdysteroid titer is also shown according to Kiguchi and Agui, 1981 [20], and indicates the stage of epidermis (4A to 5A). The gene for *ribosomal protein L3 (rpL3*) was used as an internal control (see details in materials & methods). (B) Tissue specific expression of cuticular protein genes and preferentially expressed genes identified in epM data set. Four tissues (epidermis, fat body, hemolymph, and midgut) on fourth E1 stages were used.

To determine the detailed expression profiles of several major cuticular protein genes during the last larval molt, we used semiquantitative RT-PCR analysis of epidermal mRNAs at eight developmental stages, A-E2 of the fourth instar, F (just after the fourth ecdysis), and A (day 3) of the fifth instar (Figure 4A, [20]). The expression of most cuticular protein genes is repressed at D1 with the peak of ecdysteroid during the molt, suggesting that a high dose of ecdysteriod suppresses their expression. The expression patterns of cuticular protein genes during the molt can be grouped into four divisions: first group, expressed after the decay of ecdysteroid, as well as in the intermolt phase (*BmorCPR3*, *BmorCPR4*, *BmorCPR5*, *BmorCPR38*, *BmorCPR41*, *BmorCPR10*, *BmorCPR39*, *BmorCPG4*, *BmorCPG5 *and *BmorCPG26*); second group, expressed after the decay of ecdysteroid with their disappearance at mid fifth instar (*BmorCPR83a*, *BmorCPR91*, *BmorCPG1*, *BmorCPG8*, and *BmorCPG28*); third group, not expressed during the molt (C1-E2) (*BmorCPFL1a*); and fourth group, ubiquitously expressed throughout the fourth and fifth instars (*BmorCPR148*). We speculate that the first and fourth classes are generally involved in thickening the cuticle layer, but that the second class of dominantly expressed genes during molt are mainly involved in newly synthesized cuticle formation. The second group seems to be induced by the decay of ecdysteroid. We have already reported that the expression of the second class gene *BMCPG1 *(*BmorCPG1 *in this study) is induced by an ecdysteroid pulse and may be controlled by the FtzF1 function [26], which supports the idea outlined above. It is noteworthy that the RR-2 and CPG proteins are mainly categorized in the second class, supporting the idea that these proteins form the outer layer of the cuticle, as proposed above.

A comparison of various EST libraries showed the tissue specificity of each cuticular protein gene. Of these 92 cuticular protein genes, transcripts for 28 genes were only found in the epM library (Additional Files 1 and 4). In particular, *BmorCPR39*, *BmorCPG26*, *BmorCPG3*, *BmorCPG25*, *BmorCPG28*, and *BmorCPG27 *were included in the 50 most abundantly expressed ESTs in epM (Table 2), but were not found in any other libraries, suggesting that they are mainly involved in the construction of the newly synthesized cuticle of the epidermis. As described above, transcripts for 12 CPG proteins were only found in epM, and transcripts for another five CPG genes (*BmorCPG1*, *BmorCPG14*, *BmorCPG17*, *BmorCPG20*, and *BmorCPG29*) were predominantly found in epM (over 75% of the total ESTs). To confirm the tissue-specific expression of several CPG genes, we used RT-PCR analyses of the mRNAs of the molting stage (E1) in four different tissues (epidermis, fat body, hemocytes, and midgut) and found that all of them were abundantly expressed in the epidermis in the molting stage (Figure 4B). In contrast, several cuticular proteins were predominantly expressed in tissues other than the epidermis, and in some cases, transcripts were also found in internal organs [12]. The cuticular protein genes may be expressed in different tissues in a different manner, and their composition and functional roles in tissue formation may be divergent.

### EST clones preferentially expressed in epM

In addition to the genes for cuticular proteins, many other transcripts were preferentially found in the epM library. We identified 120 clusters (Additional File 1) using the criterion that the clone was more than threefold enriched in the epM data set compared with its occurrence in other EST libraries (over 75% of ESTs were found in the epM library). Thirteen of these clusters were represented by more than four clones in the epM library (Table 3). Nine of them were already known or homologous genes, although some of them were functionally undefined. The remaining four clusters had no sequence similarity to any known proteins. We also analyzed the epidermis-specific expression of four genes (Figure 4B) and confirmed their tissue specificity. These results suggest that comparing various EST libraries is useful in screening for tissue-specific genes.

**Table 3:** Preferentially expressed genes in epM data set compared to other *Bombyx *EST libraries.

Cluster No.	Total ESTs		Gene name	CG No.	E-value*	Score*	OtherLibrary**
**Homology with known proteins**							
21	60		CG32774	CG32774	1E-06	50.8	5 (vg4M)
23	47		CG13731	CG13731	2E-19	94.4	4(vg4M, e100, ceN)
85	10	Ejaculatory bulb protein III		CG11390	1E-20	95.9	2 (epV3, mxg)
91	10	FG10924.1 (*Gibberella zeae *PH-1)				0	
157	6	Osiris 9		CG15592	8E-22	100	1 (wdV3)
197	5	Osiris 9		CG15592	1E-09	60.5	0
205	5	CG10264		CG10264	1E-13	73.6	0
207	5	TpnC25D		CG6514	9E-48	186	0
216	4	fungal protease-specific inhibitor-F (*Bombyx mori*)					
**No homologous genes**							
38	22	no homology					5 (phe, MFB, epV3)
131	7	no homology					0
175	5	no homology					0
199	5	no homology					0

* E-value and score are calculated in *Drosophila *homologs.

**Abbreviation: ceN (compound eyes, 5th-pupa), e100 (embryo, 100 hr after fertilization), epV3 (epidermis, 5th day 3), MFB (fat body, 5th-instar larva microbe-infected), mxg (maxillary galea, 5th-instar day-3 larva), phe (pheromone gland, newly-eclosed adult), vg4M (Verson's gland, 4th molting stage), wdV3 (wing disc, spinning stage day-3)

### Proteins with putative signal peptides at their N-termini

After the translation of their respective mRNAs, the cuticular proteins are transported outside the cell to the cuticle, through the function of the signal peptides at their N-termini. We found that all the cuticular protein genes identified here encoded the putative N-terminal signal peptide sequences, as characterized by the program SignalP3.0 [31] (Additional File 1) [12]. Interestingly, this program also revealed that 290 genes in epM, other than cuticular protein genes, encode N-terminal signal peptides (Additional File 1).

Willis et al. [1] summarized the nonstructural proteins found in the cuticle into four categories: (1) pigments, (2) enzymes, (3) defense proteins, and (4) arylphorins. In the first class, we found three yellow family proteins (pigmentation genes, discussed below). In the second class, we found several enzymes that are associated with sclerotization and cuticle digestion. Although we have no evidence of their transport outside (cuticle) or inside (hemolymph) the epidermal cell, at least some of these proteins seem to be transported into the cuticle: chitinase, chitinase precursor, and four chitin-binding proteins, which could be essential for cuticle formation. We also identified the *Gasp *gene (no. 1451), which is predicted to have four type-2 chitin-binding domains. Gasp is found in the tracheae that are present in the cast cuticles [3]. Molting-fluid carboxypeptidase [32] and a dozen molting-fluid carboxypeptidases (non-serine-type peptidases) may be involved in digesting the old cuticle in apolysis. Two EST clusters (nos 331 and 1230) are predicted to contain N-terminal signal peptides and to encode novel *Bombyx *peptidase proteins, which may participate in apolysis. The insect epithelium secretes innate immunity components, antimicrobial peptides, and clip-domain serine proteases, which react with hemolymph prophenoloxidase [33-36]. Among the third-class proteins encoded in the epM data set, there are six serine proteases, which are thought to be involved in immunity (Additional File 3). Their inhibitors, Serpin (a Kazal-type serine protease inhibitor) and the Kunitz family of serine protease inhibitors are expressed at the same time (Additional File 3). In the fourth class, we found three arylphorin genes (nos 109, 114, and 239). The role of arylphorin remains unknown, although it is generally assumed to participate in sclerotization because of its high tyrosine content [1]. Apart from these four classes, we also found several genes with signal peptides at their N-termini, many of which are small-ligand-binding proteins (see below).

### Hormone metabolism and the P450 family

The epM data set contains already-known genes and putative homologous genes that encode proteins involved in ecdysteroid and steroid metabolism (Additional File 3). During the molting process, ecdysteroid is finally inactivated by enzymes [37-41]. These genes may encode enzymes that inactivate ecdysteroid to terminate molting. There are also genes putatively involved in juvenile hormone (JH) metabolism and P450 genes in the epM data set. In the list of epM clusters encoding proteins with signal peptides are many known sequences (immune proteins, enzymes involved in various metabolic pathways) and proteins with putative functional domains (JH-binding proteins and odorant-binding proteins, etc.), but their actual roles in the molting stage of the epidermis remain ambiguous.

### Modifiers of cuticular protein structure

Enzymes involved in cuticular sclerotization may be excreted into the cuticular layer. Candidate genes are also included in the epM data set. Laccase 2 is involved in cuticular sclerotization in the red flour beetle, *Tribolium castaneum *[29]. Although the sequence of *Bombyx laccase 2 *in epM (cluster no. 549) is a truncated sequence, we obtained the complete sequence with 5', 3' rapid amplification of cDNA ends and analyzed its expression dynamics in the larval molting stages. *Bombyx laccase 2 *is expressed in the late molting period, when the cuticle is sclerotized (data not shown), suggesting that laccase 2 also functions in cuticular sclerotization in *Bombyx*.

Prolyl 4-hydroxylase catalyzes peptidyl-proline hydroxylation to 4-hydroxy-L-proline in collagen proteins in vertebrates [42]. Together with peptidyl-prolyl *cis*-*trans *isomerase and disulfide isomerase, it is considered to have at least three functions: (i) catalyzing the formation of intrachain and interchain disulfide bonds; (ii) as the β-subunit of collagen prolyl 4-hydroxylases; and (iii) as a chaperone that binds nascent collagen chains and prevents their aggregation [42]. Collagens are the most abundant proteins in the human body, constituting ~30% of its protein mass. In contrast, the *Drosophila *genome has only three collagen-like genes, and it is considered that these protein-modifying enzymes modify proteins other than collagen [42]. Perhaps these insect cuticular proteins are candidate targets of these protein-modifying proteins. The battery of genes included in epM supports this proposition and the general cuticle (R&R-type) proteins have 5%–18% proline in their primary sequences. Prolyl-isomerization may also play an important role in the modification of the tertiary structures of cuticular proteins.

### Small-ligand-binding proteins

The epM data set includes various putative small-ligand-binding protein genes (Additional File 3). JH-binding proteins (JHBPs) are secreted into the extracellular region, bind to JH, and are regulated by other insect hormones and nutrients [43]. JH also acts on the epidermis to prevent the ecdysteroid-induced expression of the Broad-Complex, and probably maintains larval traits [43-47]. Recently, we found that JH also regulates the larval pattern switch in the swallowtail butterfly, *Papilio xuthus *[48]. Ten JHBPs (or takeout) family proteins have been reported in *Bombyx *[49]. These proteins are assumed to bind hydrophobic ligands, although the precise ligands of the gene family are not known except for one JHBP that binds JH. Intriguingly, 16 putative JHBP genes occur in the epM data set (Additional File 3). Twelve of them are novel (nos 117, 205, 268, 337, 448, 479, 535, 646, 665, 737, 951, and 967), and five of them (nos 205, 479, 665, 951, and 967) are not included in other *Bombyx *EST data sets (Additional File 1). The sum of their clone numbers is 43. These JHBPs may be expressed and function cooperatively to maintain fourth-instar larval traits. These proteins have signal peptides at their N-termini, and the carotenoid-binding protein found in the cuticle has sequence similarity to JHBPs [50], suggesting that some JHBPs may also be secreted into the cuticle.

Unexpectedly, there are 14 genes for odorant-binding proteins in the epM data set, all of which have signal peptides at their N-termini. These proteins are known to localize to the sense organs (antennae) or pheromone glands (genitalia) in insects [51-53]. It is possible that they are associated with the sensory hairs on the epidermal surface. Although the functions of these proteins in the epidermis remain unclear, hydrophobic molecules may play important roles in molting.

A vitamin E (an antioxidant molecule)-binding protein was also isolated in this study. There have been few reports of invertebrate vitamin E (tocopherol derivatives) or α-tocopherol, which is the form mainly synthesized and accumulated in *D. melanogaster *[54]. Some tocopherol-metabolizing enzymes also occur in epM, so tocopherol may be synthesized and function as an antioxidant in the epidermis of *B. mori*.

There are some putative small-ligand-binding proteins that retain their N-terminal signal peptides. Genes for putative cellular retinaldehyde-binding proteins (seven genes) and FK506-binding proteins (two genes) are also included in the epM data set. Cellular retinaldehyde-binding protein (CRABP) has been reported to interact with all-*trans *retinoic acid, which is a structural derivative of ecdysone [55]. The overexpression of CRABP leads to the inhibition of growth in the *Bombyx *cultured cell line, BmN [55]. The epM data set also includes genes encoding FK506-binding protein (FKBP) [56], which is a member of the *Drosophila *protein family, FKBP39, and binds the immunosuppressive drug FK506. Recently, a member of the FKBP39 family was included in the ecdysone receptor and putative JH receptor Methoprene-tolerant complex [57]. Although the target substance is as yet unknown, these proteins may modify the ecdysone/JH signal cascade.

### Transcription factors

The epM data set includes 17 transcripts for genes encoding proteins homologous or similar to known transcription factors or nucleic-acid-binding proteins (Figure 1B, Additional File 3). They correspond to about 1.2% of the total nonredundant clusters. Three clones are *modifier of mdg4*, encoding the BTB/POZ domain protein of *Bombyx*, which is predicted to encode a zinc-finger protein. However, because this gene is also found in many other libraries, it may be expressed ubiquitously. Consistent with this observation, the Modifier of mdg4 protein functions with chromatin proteins in many types of tissue in *Drosophila *[58]. Two proteins known to act in the Notch pathway, E(spl)mbeta and Suppressor of Hairless (Su(H)), are also found in epM. The Notch pathway is implicated in the process of lateral inhibition and is required for the epidermal/neural fate decision in the stereotyped patterning of the sensory organs on the epidermis. Su(H) and proteins of the E(spl) complex are required for the epidermal fate decision in *Drosophila *[59]. It is noteworthy that we found a homologue of *Drosophila drumstick *(*drm*) in epM, which encodes a zinc-finger protein. The Drm protein allows the accumulation of Bowl, a zinc-finger protein whose mRNA is ubiquitously expressed, by sequestering Lines, which otherwise reduces the abundance of Bowl by binding directly to it. The patterning across the dorsal epidermis of the *Drosophila *larva is organized by the regulation of *drm *expression by Hedgehog and Wingless, which are secreted from adjacent sources flanking the parasegment boundary [60]. The inclusion of *drm *in the epM data set implies that the Drm/Lines/Bowl regulatory cassette may also function in patterning the larval epidermis of the silkworm. The gene for *slow border cells *encodes a protein that functions in choriogenesis in both *Bombyx *and *Drosophila *[61]. Intriguingly, there are features common to choriogenesis and cuticle formation. Both of them involve the formation of rigid exoskeletons with excreted proteins. This transcription factor may be a central regulator or maintain the identity of the integument in both choriogenesis and cuticle formation. These proteins and/or some hormonal regulators cooperatively specify the integument as the larvae periodically shed their egg cases.

### Pigmentation genes

The epM data set includes transcripts for nine genes encoding proteins homologous or similar to known pigmentation genes (Figure 1B, Additional File 3). In the silkworm larval body, three types of coloration are typically observed: black (probably melanin), white (probably uric acid and pteridine), and brown (probably ommochrome) [62].

#### Melanin biosynthesis

*Bombyx mori *larvae have black markings on their dorsal integuments (segments 2, 5, and 8 of the +^p ^strain and in all segments of the p^S ^strain). Because we prepared RNA from the p^S ^strain, which shows bold black stripes as the larval markings, the epM library was expected to contain several melanin synthesis genes. Six genes (encoding *phenylalanine hydroxylase *[*PAH*], *tyrosine hydroxylase *[*TH*], *yellow*, *yellow-f*, *yellow-f2*, and *tan*) are involved in the melanin synthesis pathway. In the larvae of the swallowtail butterfly, *P. xuthus*, both *TH *and *yellow *are associated with the stage-specific black larval markings [48,63,64], suggesting that these genes are associated with the *Bombyx *black markings. In contrast, PAH is a rather ubiquitous enzyme in insect tissues [65], and is broadly expressed in the larval epidermis of *P. xuthus *[66]. In *D. melanogaster*, Yellow-f and Yellow-f2 act as dopachrome-conversion enzymes downstream from dopa [67], and Tan acts as an N-β-alanyl-dopamine hydrolase, which converts N-β-alanyl-dopamine to dopamine [68]. These proteins may also function in melanin synthesis and/or black pattern regulation.

#### Ommochrome biosynthesis

Two genes, *cinnabar *and *ruby*, are associated with the ommochrome biosynthetic process. Cinnabar acts as a kynurenine 3-monooxygenase, which catalyzes the oxidation of kynurenine to 3-hydroxykynurenine [69]. Ruby is involved in vesicle trafficking [70]. Both proteins only affect eye coloration in *Drosophila*. Recently, ommochrome synthesis proteins have been associated with the adult wing coloration of the butterfly *Heliconius *[71,72]. *cinnabar *expression is associated with the forewing band, regardless of the pigment color [71]. Because ommochrome is abundant in the larval epidermis of *Bombyx*, these proteins may be involved in the larval brown coloration, although loss of function of *cinnabar *results in egg and eye coloration but not in larval body coloration [73].

#### Uric acid biosynthesis

The epM library contains one clone of the *rosy *gene. Rosy acts as a xanthine dehydrogenase, which is involved in uric acid synthesis. In *Bombyx *larvae, its mutation results in oily skin color [74,75]. Two xanthine dehydrogenase genes are highly expressed in the Malpighian tubules, fat body, and midgut [75]. Our results suggest that xanthine dehydrogenase functions in de novo uric acid synthesis in the epidermis.

## Conclusion

By exhaustive sequencing and the analysis of full-length cDNA clones, we identified 1,380 putative genes expressed in the silkworm epidermis during the larval molt. Ninety-two of these encode putative cuticular proteins, and a comparison of the epM and epV3 ESTs revealed that the expression of the cuticular protein genes is markedly different in the molting and intermolt periods. Compared with other EST data sets, we identified 13 genes preferentially expressed in the epidermis during the molt. We identified 290 other genes with signal peptide sequences, in addition to the cuticular protein genes, suggesting that some of them play novel roles in cuticle formation and the molt. In this study, we catalogued the genes expressed in the epidermis during the larval molt, which should be helpful in gaining an overview of cuticle formation and the insect molt.

## Methods

### Experimental animals and developmental stages

The laboratory silkworm stocks +^p ^(normal) and their sib-mating strain, p^S ^(Striped) were reared on the artificial diet Silkmate 2 M (Kyodo Shiryo, Yokohama) under a 16 h: 8 h L:D photoperiod at 25°C. The staging of molting period was based on the spiracle index, which represented the characteristic sequence of new spiracle formation [76]. Based on the visible characteristics, 10 morphological larval stages (A, B, C1, C2, D1, D2, D3, E1, E2 [A-E, fourth instar larva] and F [fifth]) could be distinguished, which are referred as the spiracle index. C1 is a start stage of the fourth molting, C2 is a peak stage for the ecdysteroid titre and F is just after the molt [76]. The newly molted fourth and fifth instar larvae were segregated immediately after the onset of the photophase (this day was designated day 0).

### Construction and sequencing of the full-length cDNA library

We dissected epidermal tissues from dorsal integument of segments 5, 6 of the silkworm stocks +^p ^and the sib-mating strain, p^S^. The attached muscle, fat body and Verson's glands were removed by forceps from epidermal tissues. Total epidermal RNAs of each molting stages (C1, C2, D1, D2, D3, E1, E2 and F; 8 stages) were isolated from one larva of each silkworm strain, using TRI reagent (Sigma) according to the manufacturer's instructions. Total RNAs (3 μg each) of 8 molting stages (16 samples) were mixed and subjected to full-length cDNA library construction, which was performed by Hitachi Science Systems, Ltd. Japan. The construction of the full-length cDNA library was made by a G-capping method previously reported by Ohtake et al., 2004 [20], which enables relatively long insertions. We named this library as epM (fourth instar epidermis in Molting). After construction of the cDNA library, the library quality was evaluated by 96-clone test sequencing (Library size, 1.54 × 10^5^; Ratio of insert-including bacterial clone, 79%; Ratio of full-length cDNA insert, 76%; Average of insert length, 0.9 kb). Judgment of the full-length cDNA was followed with Ohtake et al., 2004 [20]. More than 10,000 cDNA clones were picked up randomly from the epM library, and obtained a sequence of ~650 nucleotides from the 5' end of each cDNA in average.

### Sequence Analysis and characterization of epM data set

The sequences more than 300 nucleotide length were taken as EST in the epM dataset. Nonredundant EST clusters were identified by clustering with EST clones of the other libraries with the criterion previously described [19]. By comparison of the deduced amino acid sequences with public protein databases InterProScan: [77], gene classification was assigned by using a criterion of homology of > 30% identity in a sequence > 100 amino acids as well as an *E *value lower than e^-15 ^in a BLAST search (previously described [19]). Gene category was classified with GO term [78] or with FlyBase GO classification [21]. Identification and naming of cuticular protein genes were referred as Futahashi et al., 2008 [12], and cuticular protein genes were classified as structural molecule activity (GO:0005198) and structural constituent of cuticle (GO:0042302) in GO term. SignalP 3.0 [31] was used for signal peptide prediction.

### Reverse transcription polymerase chain reaction (RT-PCR) analyses

To determine expression levels of cuticular protein genes, total epidermal RNA from fourth instar stages (A, B, C1, D1, E1, E2) and fifth (F and day3) were extracted. To detect tissue-specificity of EST clones, total RNA of fourth instar (stage E1) epidermis, fat body, hemocytes, and midgut were extracted. Total RNA was treated with RNase-free DNase (TaKaRa), and 1 μg of total RNA was reverse transcribed using First-Strand cDNA Synthesis Kit (Amersham). The primer sets were described in Additional File 5. The PCR conditions used were 96°C for 2 min followed by 30 (or 33) cycles of 96°C for 15 sec, 50°C (or 52°C) for 15 sec, and 72°C for 1 min. The reactions were kept at 72°C for 1 min after the last cycle. The gene for the ribosomal protein L3 (rpL3) [79] that is expressed constitutively in the cell was used as an internal control for normalization of equal sample loading.

## Abbreviations

CPF: cuticular protein(s) with 44-amino acid motif; CPFL: CPF-like protein; CPG: cuticular protein glycine-rich; CPH: cuticular protein hypothetical; CPR: cuticular protein(s) with the R&R Consensus; CPT: cuticular protein(s) with a Tweedle motif; EST: expressed sequence tag; FKBP: FK506 binding protein; GO: gene onthology; HCS: head capsule slippage; JH: juvenile hormone; JHBP: JH binding protein; R&R Consensus: Rebers and Riddiford Consensus; RR-1, RR-2, and RR-3: three class of CPR proteins; RT-PCR: reverse transcription and polymerase chain reaction; 20-E: 20-hydroxyecdysone.

## Authors' contributions

SO carried out the molecular genetic studies, participated in the construction of EST library and performed RT-PCR analysis, and wrote the manuscript; RF performed sequence data analysis and wrote the manuscript; TK wrote and assisted preparation of manuscript; KM directed the sequencing and data analysis; HF directed the experimental work on EST construction and data analysis and completed the manuscript. The project was conceived and coordinated by KM and HF. All authors have read and approved the final version of this manuscript.

## Supplementary Material

Additional File 1 List of nonredundant gene sequence and its number of epM data set. 1–1380 nonredundant sequences in epM were listed. The column of "Other libraries" indicates the numbers of the corresponding sequence found in the silkworm EST libraries constructed by Japanese groups, other than the epM library. "epM/total" represents the percentage of EST numbers of epM to total silkworm EST data set. DmGene represents an orthologous gene in *Drosophila melanogaster* Click here for file

Additional File 2 List of identified putative isoforms or premature transcript. Click here for file

Additional File 3 List of characteristic gene groups identified in epM dataset. Click here for file

Additional File 4 List of identified cuticular proteins and comparison with epV3 library. The number in epM and epV3 columns indicates the genes expressed in the respective data sets. Click here for file

Additional File 5 Oligonucleotide primers used for analysis of tissue- and stage-specific expression of selected genes. Click here for file
