# Supplementary material for: Catalogue of epidermal genes: Genes expressed in the epidermis during larval molt of the silkworm Bombyx mori
Source: BMC Genomics. 2008 Aug 22;9:396. doi: 10.1186/1471-2164-9-396 (PMC2542385; doi:10.1186/1471-2164-9-396)
Supplement: Additional File 3 — List of characteristic gene groups identified in epM dataset. [file 1471-2164-9-396-S3.doc]

ClusterNo totalESTs Gene Name CG No. E-value Score

Chitin-binding structural protein, non-RR type (5 genes)

45 18 Chitin binding Peritrophin-A CG10287 473 2.00E-133

202 5 Chitin binding Peritrophin-A CG4778 283 8.00E-77

319 4 Chitin binding Peritrophin-A CG8192 135 2.00E-32

439 2 Chitin binding Peritrophin-A CG17058 330 6.00E-91

1248 1 Chitin-binding, domain 3 CG15786 153 8.00E-38

Chitin metabolism (12 genes)

92 10 Glycoside hydrolase CG32209 419 9.00E-118

203 5 Glycoside hydrolase CG7997 325 2.00E-89

208 5 Chitinase - - -

271 3 Glycoside hydrolase CG9701 265 2.00E-71

283 3 Chitinase CG1780 265 2.00E-71

336 2 Glycoside hydrolase CG5731 172 2.00E-43

410 2 Chitin binding CG13643 58.9 3.00E-09

534 1 Glycoside hydrolase CG9701 226 9.00E-60

573 1 Glycoside hydrolase CG9307 371 3.00E-103

1146 1 Chitinase CG18140 191 4.00E-49

1328 1 galactosyltransferase CG11780 249 9.00E-67

1372 1 Glycoside hydrolase CG18140 55.1 4.00E-08

Cuticle Protein Structure Modifier (5 genes)

351 2 Peptidyl-prolyl cis-trans isomerase CG2852 248 2E-66

549 1 Laccase2 CG30437 343 4.00E-95

677 1 Prolyl 4-hydroxylase CG31022 149 2.00E-36

724 1 Disulfide Isomerase CG8983 246 6.00E-66

1279 1 Peptidylprolyl isomerase CG9847 304 4E-83

Excreted Protease (16 genes)

97 9 prophenoloxidase activating enzyme CG3066 107 7.00E-24

145 7 trypsin-like serine peptidase CG5390 277 1.00E-74

190 5 trypsin-like serine peptidase CG5390 83.6 1.00E-16

172 6 serine carboxypeptidase - - -

329 2 cathepsinD CG1548 294 4.00E-80

331 2 serine carboxypeptidase CG4572 130 5.00E-31

344 2 metallopeptidases CG6763 147 5.00E-36

452 2 cathepsinB CG10992 258 3.00E-69

517 1 trypsin-like serine peptidase - - -

739 1 serine carboxypeptidase CG4572 174 4.00E-44

869 1 trypsin-like serine peptidase CG3066 141 4.00E-34

1013 1 trypsin-like serine peptidase - - -

1041 1 cysteine peptidase CG32816 89.7 2.00E-18

1111 1 Zinc carboxypeptidase CG3108 167 5.00E-42

1230 1 Peptidase S28 CG9953 221 4.00E-58

1298 1 trypsin-like serine peptidase CG4998 92.8 2.00E-19

Hormone and lipid metabolism (19 genes)

89 10 Cytochrome P450 CG11715 313 1.00E-85

291 3 Cytochrome P450 CG5137 99.8 2.00E-21

364 2 steroid dehydrogenase CG13284 205 2.00E-53

368 2 sterol carrier protein CG17320 228 4.00E-60

445 2 Di-trans-poly-cis-decaprenylcistransferase - - -

318 3 farnesoic acid O-methyltransferase CG10527 176 1.00E-44

531 1 farnesoic acid O-methyltransferase CG10527 58.5 3.00.E-09

544 1 3-oxoacid CoA-transferase CG1140 211 3.00E-55

799 1 Cytochrome P450 CG8587 285 2.00E-77

872 1 farnesyl pyrophosphate synthase CG12389 160 5.00E-40

943 1 steroid dehydrogenase CG7113 160 2.00E-40

1038 1 estradiol 17-beta-dehydrogenase CG11151 87.4 4.00E-18

1139 1 acyltransferase, lipid metabolism CG5278 194 4.00E-50

1179 1 esterase CG4390 174 2.00E-44

1203 1 haloalkane dehalogenase - - -

1233 1 Carboxylesterase, type B CG4757 229 9.00E-61

1270 1 Terpenoid synthase CG10585 178 2.00E-45

1312 1 steroid dehydrogenase CG7113 189 7.00E-49

1359 1 fatty acid elongase CG2781 278 2.00E-75

Protease Inhibitor (20 genes)

44 18 Protease Inhibitor, Kazal-type CG1220 66.2 2.00E-11

110 9 serpin CG9453 170 1.00E-42

166 6 Cathepsin propeptide inhibitor - - -

220 4 Protease Inhibitor, Kazal-type CG8369 52.4 2.00E-07

357 2 serpin CG10913 103 1.00E-22

366 2 Protease Inhibitor, Kazal-type - - -

391 2 serpin CG6680 105 2.00E-23

453 2 serpin CG1342 71.2 5.00E-13

458 2 serpin CG9334 135 2.00E-32

464 2 serpin CG9460 62.4 3.00E-10

475 2 Protease Inhibitor, Kunitz type CG12540 83.6 1.00E-16

502 1 Cathepsin propeptide inhibitor CG12163 47 7.00E-06

512 1 serpin - - -

611 1 Protease Inhibitor, Kunitz type CG33103 103 9.00E-23

628 1 Protease Inhibitor, Kunitz type - - -

848 1 Protease Inhibitor, Kazal-type CG32354 191 5.00E-49

889 1 Protease Inhibitor, Kunitz type CG16712 62.4 5.00E-11

1122 1 Protease Inhibitor, Kunitz type CG2816 71.6 2.00E-13

1162 1 Protease Inhibitor, Kazal-type - - -

1107 1 Cathepsin propeptide inhibitor - - -

Small ligand binding (41 genes)

117 8 JH-binding CG14457 201 5.00E-52

121 8 JH-binding (An0921) CG11852 68.2 5.00E-12

205 5 JH-binding CG2650 73.6 1.00E-13

215 4 JH-binding (Brp2095) CG10407 102 3.00E-22

268 3 JH-binding - - -

337 2 JH-binding CG10407 95.1 4.00E-20

448 2 JH-binding CG10407 90.9 7.00E-19

479 2 JH-binding CG14457 51.6 4.00E-07

535 1 JH-binding CG10407 95.9 2.00E-20

646 1 JH-binding CG11854 63.2 1.00E-10

661 1 JH-binding (wdS30639) CG10407 99 2.00E-21

665 1 JH-binding - - -

737 1 JH-binding CG11852 60.8 6.00E-10

805 1 JH-binding (JHBP) - - -

951 1 JH-binding CG2650 88.6 3.00E-18

967 1 JH-binding CG10407 62.4 3.00E-10

40 20 Odorant-binding CG11390 102 2.00E-22

56 14 Odorant-binding CG11390 122 2.00E-28

85 10 Odorant-binding CG11390 85.9 1.00E-17

156 6 Odorant-binding CG11390 96.7 1.00E-20

196 5 Odorant-binding CG8462 73.6 8.00E-14

198 5 Odorant-binding CG11390 114 6.00E-26

250 3 Odorant-binding CG30172 70.9 7.00E-13

376 2 Odorant-binding CG11390 111 3.00E-25

451 2 Odorant-binding - - -

462 2 Odorant-binding CG9358 64.3 4.00E-11

754 1 Odorant-binding CG11390 85.9 2.00E-17

762 1 Odorant-binding CG11390 106 1.00E-23

971 1 Odorant-binding - - -

1063 1 Odorant-binding CG9358 79.3 1.00E-15

833 1 VitaminE-binding CG12926 77.4 6.00E-15

278 3 Cellular retinaldehyde-binding CG2663 235 2.00E-62

430 2 Cellular retinaldehyde-binding CG2663 102 2.00E-22

592 1 Cellular retinaldehyde-binding CG2663 82 3.00E-16

757 1 Cellular retinaldehyde-binding CG3823 162 1.00E-40

1161 1 Cellular retinaldehyde-binding CG3823 143 1.00E-34

1284 1 Cellular retinaldehyde-binding CG10237 244 2.00E-65

1323 1 Cellular retinaldehyde-binding CG10026 193 9.00E-50

380 2 FK506-binding CG11001 181 4.00E-46

1218 1 FK506-binding CG1847 142 2.00E-34

Transcription Factor Activity Genes (17 genes)

289 3 modifier of mdg4 CG32491 2E-46 182

367 2 Limpet CG32171 1E-111 399

416 2 Limpet CG32171 1E-25 113

491 2 bunched CG5461 8E-24 107

513 1 CG7839 CG7839 2E-15 79.3

612 1 Suppressor of Hairless CG3497 4E-66 247

639 1 E(spl) region transcript mbeta CG14548 2E-30 128

733 1 CG4914 CG4914 2E-28 120

766 1 anterior open CG3166 5E-47 184

801 1 nautilus CG10250 3E-21 98.6

900 1 drumstick CG10016 1E-36 149

1061 1 MTA1-like CG2244 2E-37 151

1082 1 CG5343 CG5343 5E-105 377

1153 1 slow border cells CG4354 4E-30 128

1238 1 apterous CG8376 3E-45 178

1250 1 DP transcription factor CG4654 2E-12 69.7

1354 1 no ocelli CG4491 1E-36 150

pigmentation genes (9 genes)

Melanin biosynthesis

70 12 yellow CG3757 3E-125 445

266 3 tan CG12120 1E-57 219

275 3 pale (tyrosine hydroxylase) CG10118 2E-52 202

559 1 yellow-f CG18550 9E-18 87

654 1 yellow-f2 CG8063 4E-16 81.6

786 1 Henna (phenylalanine hydroxylase) CG7399 1E-79 292

Ommochrome biosynthesis

1012 1 cinnabar CG1555 9E-28 119

1027 1 ruby CG11427 1E-30 129

Uric acid biosynthesis

1207 1 rosy (xanthine dehydrogenase I) CG7642 2E-75 278

**Additional File 3 Characteristic gene groups identified in epM dataset**

* E-value and score are calculated in *Drosophila* homologs.
